# Self-compassion in context: a reflexive thematic analysis of migrant domestic workers’ experiences in Singapore

**DOI:** 10.1080/17482631.2026.2617449

**Published:** 2026-01-19

**Authors:** Yeoh Lai Lin Rachel, Barlas Joanna

**Affiliations:** a School of Social and Health Sciences, James Cook University, Singapore, Singapore; b Margaret Roderick Centre for Mental Health Research, James Cook University, Douglas, QLD, Australia

**Keywords:** Self-compassion, migrant domestic workers, reflexive thematic analysis, sociocultural context, well-being

## Abstract

**Objectives:**

Migrant Domestic Workers (MDWs) face adverse living and working conditions that place them at a heightened risk of poor mental health outcomes. While prior research has examined MDWs’ coping strategies and resilience, little is known about how they understand and engage in self-compassion, an intrapersonal process strongly linked to mental well-being. This study aimed to examine how MDWs conceptualize self-compassion and the sociocultural factors that influence their capacity to practice it.

**Methods:**

Semi-structured interviews were conducted with ten female Filipino and Indonesian MDWs living in Singapore recruited through purposive and snowball sampling. Data were analyzed using reflexive thematic analysis.

**Results:**

Three interrelated themes were generated: (1) grounding self-compassion in self-worth and identity, (2) contextual conditions shaping self-compassion, and (3) enacting self-compassion in everyday life. These themes illustrate the complex interplay between participants’ internalized self-perceptions, cultural narratives, and structural conditions that shape their engagement with self-compassion.

**Conclusions:**

The findings contribute to a more nuanced understanding of self-compassion in marginalized caregiving populations. They advocate for the need to address structural determinants of MDWs’ well-being and to develop culturally sensitive psychoeducation and interventions.

## Introduction

Singapore hosts approximately 286,300 Migrant Domestic Workers (MDWs) and these individuals hold a work permit that binds them to a single employer (Ministry of Manpower, 2024a). They are predominantly female and typically live in their employer’s household. Their roles encompass various domestic duties including housekeeping, childcare, and elderly care. Many leave their home countries to seek employment in wealthier nations, motivated by higher earnings and the opportunity to secure an improved future for themselves and their families (Yeoh & Huang, 2000). In Singapore, the majority of MDWs originate from Indonesia and the Philippines, with the remaining nationalities primarily from countries such as Myanmar, Sri Lanka, India, Thailand, and Bangladesh (Transient Workers Count Too, 2011).

### Challenges faced by MDWs

MDWs in Singapore are excluded from the Singapore Employment Act, which safeguards against unfair employment conditions (Ministry of Manpower, 2024b). As a result, they lack adequate labour protection which creates a significant power imbalance favoring employers and can lead to adverse working and living conditions for MDWs (Parreñas et al., 2021). Research has documented various forms of mistreatment that MDWs face in Singapore. Examples noted across the literature include underpayment or salary withholding, lack of privacy, threats of repatriation, denial of food, excessive working hours, restricted communication, racism, harassment, confinement, and verbal, physical, and sexual abuse (Ha et al., 2018; Kaur-Gill & Dutta, 2021; Malhotra et al., 2013; Ueno, 2009; Yeoh & Huang, 2009). Beyond workplace hardships and exploitation, MDWs also experience multiple migration-related stressors, including a lack of belonging to their host country, disconnection from their home country, discrimination, alienation, financial obligations to support their families, and emotional strain of separation from their communities (Lai, 2011; Straiton et al., 2017; Van Bortel et al., 2019; Zainal & Barlas, 2022).

Parreñas and colleagues (Parreñas & Silvey, 2018; Parreñas et al., 2021a, 2021b), in a series of studies, argue that these migration and employment conditions structurally produce and sustain precarity. With families to support and limited earning opportunities in their home countries, MDWs often have no alternative but to seek employment overseas. Migration for domestic work frequently incurs debt through imbalanced and exploitative recruitment processes which, combined with contractual restrictions, limited labour mobility and long-term settlement pathways in host countries, keeps them in a state of precarity.

Thus, it is unsurprising that MDWs are vulnerable to poor mental health outcomes. Marginalised migrant communities often experience higher rates of mental health issues (Bhugra & Gupta, 2010), and research has shown that MDWs frequently experience stress (Anjara et al., 2017; van der Ham et al., 2014) and are more susceptible to mental health problems (Lau et al., 2009). Furthermore, MDWS often lack access to health and social care services (Dutta et al., 2018; Ho et al., 2022), further exacerbating the risk of poor health and diminished quality of life. A study by HOME (2015) involving 670 MDWs in Singapore found that approximately one in four experienced psychological distress and that MDWs were twice as likely to develop mental health problems compared to local residents.

### Resilience and coping among MDWs

Recent research has explored broad concepts of resilience and coping among MDWs in Singapore. Identified resources include social support from peers, maintaining connections with family back home, participating in religious and community activities, engaging with social services, reframing challenges, employing endurance and acceptance as coping strategies, and cultivating positive relationships with employers to avoid potential conflicts (Van Bortel et al., 2019; van der Ham et al., 2014; Zainal & Barlas, 2022). However, further research is needed to identify effective coping strategies that enhance mental health and resilience (Terrighena & Barron, 2020). This study aimed to contribute to existing knowledge by exploring self-compassion as a specific component of coping.

### Self-compassion as adaptive coping

Neff (2016) operationalized self-compassion as a construct involving three key components: responding to personal suffering with kindness rather than judgement, understanding suffering as part of the human experience rather than feeling isolated, and approaching suffering with mindfulness rather than over-identification. Self-kindness includes recognising personal imperfections while treating oneself with warmth and care, which can help counteract the belief of being unworthy of acceptance. Recognising common humanity involves understanding that challenges are part of the universal human experience and countering the belief that personal difficulties are unique, which fosters a sense of abnormality and isolation. By acknowledging that suffering is part of common humanity, individuals can extend empathy and kindness towards themselves instead of succumbing to self-pity, allowing them to feel supported and cared for (Neff, 2023). She posits that mindfulness allows someone to offer compassion, preventing overidentification with suffering that can lead to becoming consumed by it and losing the perspective needed to extend care to oneself.

Neff (2023) further defined self-compassion as acknowledging and responding to one’s own suffering with understanding and kindness and taking steps to alleviate that suffering. In this way, hardship is seen as a foundational context within which self-compassion emerges. Self-compassion can also involve asserting personal needs and finding empowerment to protect personal interests, pursue desires, and seek positive changes.

Self-compassion is a valuable component of coping, as research has established robust links between self-compassion and well-being (Phillips & Hine, 2021; Zessin et al., 2015). A meta-analysis by Ewert et al. (2021) found a positive association between self-compassion and adaptive coping. Greater self-compassion has been linked to reduced psychopathology in various systematic reviews (e.g. Hughes et al., 2021; MacBeth & Gumley, 2012; Marsh et al., 2018), influenced by factors such as reduced negative automatic thoughts and an increase in positive thinking (Yip & Tong, 2021), enhanced emotional regulation skills (Inwood & Ferrari, 2018), reduced avoidance of challenging emotions (Yela et al., 2022), and reduced preoccupation with negative emotions (Miyagawa & Taniguchi, 2020).

Furthermore, meta-analyses have indicated that self-compassion interventions can cultivate self-compassion and improve well-being (Ferrari et al., 2019; Kirby et al., 2017). Such interventions have also been found to reduce shame (Johnson & O’Brien, 2013), lower depression levels (Shapira & Mongrain, 2010), alleviate stress and burnout, and strengthen resilience among workers across professions (Delaney, 2018; Eriksson et al., 2018).

### Self-compassion across cultures

Cultural norms appear to shape how individuals perceive and respond to measures of self-compassion (Zhao et al., 2021). The Self-Compassion Scale (SCS; Neff, 2003b) is the most widely used measure of self-compassion across cultures (Neff et al., 2019). It was designed to measure six factors: self-kindness, self-judgement, common humanity, isolation, mindfulness, and over-identification, as outlined by Neff (2016). Despite the widespread use of the SCS, the six-factor model underlying it has shown inconsistent replication across cultural contexts, including in samples from Japan and China (Neff et al., 2019; Zeng et al., 2016). This suggests that self-compassion may be conceptualised differently across cultural contexts (Montero-Marin et al., 2018). For instance, a study on oncology nurses in Turkey found that they viewed self-compassion as encompassing self-understanding, self-protection, valuing oneself, setting aside time for oneself, self-tolerance and being a “good mother” towards oneself, and as a quality that in turn facilitates compassion toward others (Serçe Yüksel et al., 2022). A study of Canadian high school adolescents found that they saw self-compassion as prioritising themselves over others, maintaining positivity during hardships, engaging in pleasurable activities, connecting positively with others, working towards self-improvement, making themselves attractive to others, practising self-acceptance, and experiencing emotional balance (Klingle & Van Vliet, 2019). Although the SCS is widely used, its potential limitations in capturing self-compassion across cultures highlight the need for more qualitative research into conceptualisations of self-compassion, as called for by Tóth-Király and Neff (2021).

Within Southeast Asia, where this study is situated, research on self-compassion remains limited and no studies have investigated self-compassion from the ground up using qualitative methodology. Quantitative studies provide some insight, suggesting that self-compassion is similarly mobilised in response to hardship (Sambrana, 2023) but may be expressed differently in collectivistic cultures such as Philippines (Concha, 2025) and Indonesia (Kotera et al., 2022). While Filipino college students demonstrated higher compassion for others than for themselves, a symbiotic and mutually beneficial relationship between self-compassion and compassion for others emerged under conditions of horizontal collectivism, which emphasise shared responsibility and interpersonal equity (Concha, 2025). In Indonesia, baseline levels of self-compassion among students were higher than that of UK students, interpreted as reflecting reduced emphasis on individualistic success and stronger orientation toward social belonging (Kotera et al., 2022). Taken together, these findings indicate that collectivistic values of maintaining social harmony and fulfilling relational obligations seem to shape how self-compassion is expressed. Caring for the self is often viewed as relationally embedded and culturally responsible, rather than as an individualistic form of self-advocacy. At the same time, across both Philippines and Indonesia, structural pressures, such as socioeconomic strain or intense academic demands, appear to undermine the capacity for self-kindness (Kotera et al., 2022; Sambrana, 2023).

### The present study

Against this backdrop, the present study explores how MDWs in Singapore understand and enact self-compassion within their unique sociocultural and occupational contexts. Investigating this is important, as MDWs occupy a unique position where their workplace is typically also their residence, resulting in increased and imbalanced interactions with employers and, thus, heightened vulnerability to workplace stressors such as mistreatment and abuse. These stressors can have an adverse impact on mental health, and findings from this study could inform the design of specific compassion-based self-help and facilitator-led interventions aimed at improving health outcomes for this at-risk population. Furthermore, by exploring the distinct vantage point of MDWs, this study may offer insights into how sociocultural factors and socioeconomic status affect the level, functioning, and benefits of self-compassion, an area that warrants further research (Neff, 2023).

The present study aimed to explore the perceptions, experiences, and practices of self-compassion among MDWs in Singapore. The key research questions were as follows: How do MDWs conceptualise self-compassion? What do MDWs perceive as barriers and facilitators to the practice of self-compassion?

## Method

This study was underpinned by a constructivist approach that posits that MDWs construct their understanding of self-compassion through their experiences, perspectives, and interactions. By valuing individual perspectives as key insights, this approach captures the nuances of MDWs’ lived experiences and provides a deeper understanding of their perceptions and experiences of self-compassion. Such insights may be overlooked by quantitative measures that cannot fully capture the sociocultural complexity of how MDWs perceive and eexperience self-compassion.

### Participants

Ten female MDWs were recruited, comprising eight from Indonesia and two from the Philippines. The demographic details are presented in Table I. Inclusion criteria required participants to hold a valid work permit and have at least two years of experience as an MDW in Singapore. Basic proficiency in English was also required as the interviews were conducted in English. To ensure that participants had an appropriate level of English proficiency, they were asked to explain the participant information sheet and informed consent form before providing written consent and answering the interview questions. The final sample size of ten participants was determined pragmatically, considering the narrow focus of the research questions, the use of individual interviews as the data collection method, and the focus on exploring specific cultural interpretations and definitions of self-compassion (Braun & Clarke, 2021). This is in line with the sample size used by a similarly narrow exploration of self-compassion within a specific subgroup (Bohadana et al., 2021).

**Table I.** Participant demographics.

Participants (pseudonyms have been used)	Country of origin	Age	Number of years in Singapore
Wendy	Indonesia	41	14
Melissa	Indonesia	38	12
Laura	Indonesia	38	14
Yasmin	Indonesia	42	18
Ruth	Indonesia	31	8
Frieda	Philippines	38	12
Rosalind	Indonesia	39	17
Hannah	Indonesia	41	10
Sheryl	Indonesia	45	14
Janelle	Philippines	51	17

### Procedure

Ethical approval was obtained from the James Cook University Human Research Ethics Committee and all participants provided written informed consent to participate. Participants were recruited between February 2024 and April 2024 using purposive and snowball sampling strategies. Recruitment materials were distributed through a local non-profit organisation providing community support to migrant workers and via a Facebook page shared by social contacts. This approach was chosen to reach a diverse range of MDWs living and working in Singapore while ensuring participants could self-select into the study based on interest and eligibility.

Individual semi-structured interviews were conducted either face-to-face or virtually. The interviews began with an introduction to the study, provision of the information sheet, and collection of written informed consent. Interviews lasted between 37 minutes and 65 minutes and were audio-recorded. A semi-structured interview schedule (Supplement A) was developed to explore participants’ understandings and experiences of self-compassion in the context of their work and daily lives and with reference to existing qualitative studies on self-compassion (e.g. Dunkley‐Smith et al., 2021; Pauley & McPherson, 2010). It was piloted with one migrant domestic worker to ensure cultural relevance and clarity of language. The interview schedule included open-ended questions designed to elicit participants’ reflections on how they respond to personal challenges, show kindness to themselves, and understand compassion in relational and cultural contexts. Basic demographic information (i.e. age, country of origin, and number of years working in Singapore) was gathered to provide context for the participants’ narratives. The participants received a $20 token of appreciation to cover their time and travel expenses.

Participants’ interviews were transcribed verbatim with the assistance of Otter AI software, de-identified, and each participant was assigned a pseudonym to ensure confidentiality. They were invited via email to review their de-identified interview transcripts in order to verify their accuracy. Six out of ten participants responded, with three identifying clarifications, changes, or elaborations. This feedback provided an opportunity to further explore topics or insights that might not have been fully captured during the initial interview. New information from this process was included as additional data. One participant submitted an amended transcript, having utilised a grammar-checking tool to enhance readability, which resulted in a 60% reduction in word count. Since no other participant used such tools, both the original and amended transcripts were coded, and any quotes from this participant were taken from the original transcript with her consent.

### Data analysis

Reflexive thematic analysis (RTA; Braun & Clarke, 2006; Braun & Clarke, 2022) was employed to analyse the interview transcripts, allowing for deep engagement with the data to generate themes that reflect MDWs’ subjective experiences of self-compassion. This approach supports both theory-driven and data-driven analysis, capturing the nuances of participants’ lived experiences while drawing on established frameworks of self-compassion.

The transcripts were reviewed multiple times by the first author to foster familiarity and facilitate an initial understanding of the data. The first author then conducted line-by-line coding to generate preliminary codes representing specific ideas. The approach to coding was both theory-driven and data-driven by examining commonalities and cultural differences in the conceptualisation of self-compassion across transcripts. The generated codes were then grouped into subthemes and main themes, which were reviewed and refined by both the authors. This involved combining similar themes, splitting overly broad themes, and discarding themes that did not align with the data or answer overarching research questions. The main themes were then named, defined, and described in detail, with supporting examples and participant quotations as illustrations.

### Trustworthiness

Research procedures recommended by Stahl and King (2020) were followed to ensure the credibility and trustworthiness of the results. This included investigator triangulation, in which the second author reviewed a subset of transcripts coded by the first author. The first author also engaged in reflexive self-analysis through journaling and bracketing personal opinions, enhancing transparency and rigour in the analysis, while allowing for critical reflection on personal biases and perspectives.

The first author is a Chinese Singaporean trainee clinical psychologist, and her interest in self-compassion originated from clinical experience, where she observed clients often struggling with self-criticism. This motivated her to study self-compassion and Kristin Neff’s works. She frequently interacts with the MDWs employed by her family but does not employ them. These interactions provided some familiarity with the everyday realities of MDWs, which may have shaped her sensitivity to participants’ emotional expressions and cultural contexts. The second author is a White British clinical psychologist and academic. She employs an MDW and conducts research on the mental health of MDWs. Her positionality as an employer, a researcher and a non-citizen resident in Singapore introduced an awareness of privilege and potential power dynamics, prompting reflexive consideration of bias and assumptions throughout the research process. Together, the authors engaged in ongoing reflexive discussions about how their racial, cultural, and socioeconomic positions might influence the interpretation of participants’ narratives, and the representation of their voices.

## Results

Three themes and eight sub-themes were generated during the analysis. Table II summarises the themes and subthemes explored in this section.

**Table II.** Table of themes and subthemes.

Themes	Subthemes
Grounding self-compassion in self-worth and identity	The importance of self-worth Expanding purpose and identity beyond domestic work
Contextual conditions shaping self-compassion	Employer dependence and power Family obligations and self-care Marginalisation and self-worth
Enacting self-compassion in everyday life	Emotional and cognitive resilience Behavioural strategies Engaging social support

### Grounding self-compassion in self-worth and identity

Participants’ understanding of self-compassion was rooted in their emerging sense of self-worth and purpose, with two subthemes generated. First, they identified self-worth as a fundamental basis for self-compassion, suggesting that viewing themselves as worthy and significant was essential to practising compassion towards themselves. They then connected their self-worth to a broader sense of purpose and identity beyond their roles as MDWs.

#### **The importance of self-worth**

Participants explicitly connected their capacity for self-compassion to their sense of self-worth, affirming the importance of their rights, interests, and feelings and advocating for themselves by speaking up to their employers.

I want to have some freedom (…) so I speak up what I want, what I need. Like I want to go study, go out, see the world and upgrade myself. I can't just do housework and sleep forever until the end of my life. I have a life, so I express what in my mind to my employer. (Melissa)

Building self-worth also involved recognising that they should not be treated unfairly and deserved better treatment, as demonstrated in decisions to leave challenging environments. As Frieda shared, “I decided to change employer. I said, okay, I need to do this myself. I don't want to stay in this environment. It will not make me happy and I don't have peaceful mind if I stay there.”

Participants described prioritising self-interest as a form of self-compassion tied to a strong sense of self-worth. This primarily manifested in resisting pressure to provide additional financial support to families and communities in their home countries, instead establishing boundaries and choosing to spend their income according to their own needs.

Sometime we need to love ourselves. We need to count how much of our income (…) we cannot always listen to them to help them. We need to help [ourselves]. We need…we go out, we need to spend some money to buy something self-love. (Sheryl)

#### **Expanding purpose and identity beyond domestic work**

While this subtheme begins from participants’ reflections on the purpose of their work, it extends to how they derived a sense of purpose and identity beyond their MDW role. Having purpose contributed to their sense of self-worth, enabling them to view themselves as deserving of self-compassion.

Six participants identified that providing for their families back home fostered this sense of purpose. As Rosalind shared, “I proud that I can bring up [my children]. I can burden their school fee then I give them comfortable life.” Recognising these accomplishments helped the participants affirm their capabilities, making them feel worthy of self-compassion.

I achieved a lot of things in life. I'm a single mom since 2008. I bringing up my daughter and they grow up so well. They are good. I also have my family. So I deserve a reward. If nobody is going to give me a reward, I can give a reward to myself. This is one of self-compassion. (Melissa)

Furthermore, participants perceived self-compassion as essential for sustaining their ability to provide for their families, while their role as providers equally reinforced the need to be self-compassionate.

Thinking of my family (…) I have my aging mum, my children, and my granddaughter. So being kind to myself, being compassionate to myself looks like it's because of them (…) I have to take care of myself. I need to be healthy and strong. (Janelle)

Participants identified planning for the future as another source of purpose that contributed to their sense of self-worth. This included saving money for retirement, starting a business back home, and pursuing personal goals. As Hannah described, “working on my dream. That’s helped me a lot. Make me happy. Because I’m planning to have a business, I’m working on that.”

Participants described cultivating identities beyond their roles as MDWs as crucial to building self-worth. As Yasmin described, “I think it is how to show yourself you not just helper. How you're more possible and to love yourself, respect yourself, to not let other people make you down.” They described being able to appreciate their broader potential and interests by viewing themselves as more than MDWs. Many sought self-improvement through further studies, which enabled them to recognise their potential for growth and cultivate a stronger sense of self-worth. As Laura shared, “Study really helped me. Perhaps in the future, I can change job. But somehow, now, I'm very happy (…) I'm working. And also I'm studying.” Self-improvement was identified as enhancing confidence and self-worth, thus enabling participants to practice self-compassion by expressing their needs. As Melissa shared, “when we confident, we also confident to be kind to [ourselves]. When we able to communicate what happen, what we feel, what we want in a kind way.”

The participants highlighted community involvement as a way to see themselves beyond their roles as MDWs, providing opportunities for social connections, enjoyable activities, and volunteering, which fostered a sense of purpose and self-worth.

In terms of health it helps me to be nice to myself, be confident. You're not only helper; you also have something give back to community. So I always do sports, volunteers, do charity things so it makes me happy. (Frieda)

### Contextual conditions shaping self-compassion

Participants discussed how their social and occupational positioning as MDWs shaped how they understood, expressed and enacted compassion towards themselves, particularly when navigating daily challenges and structural constraints. The three subthemes reflect different contexts of their lived realities: employer dependence and power, family obligations and self-care, and marginalisation and self-worth. Each subtheme examined the challenges within these specific contexts in which self-compassion emerged.

#### Employer dependence and power

This subtheme highlighted a paradox participants often faced: the moments when self-compassion was most needed, when they felt undermined, criticised or devalued, were also the moments when it was most difficult to access. This tension revealed how external invalidation could erode and simultaneously call forth the need for self-kindness. Most participants described how experiencing threats, criticism, and scoldings fostered feelings of inadequacy, undermined self-worth, and made self-compassion challenging. As Hannah shared, “The way they talk to me, the words they used really get me down. They really made me feel guilty, really bad and shame because I couldn’t do my best.” This sense of inadequacy also led to feelings of helplessness, further hindering self-compassion.

I try my best but still have some complain. I already trying my best you know. I really trying. So what can I do more than that? (…) Then my heart make me feel down. Even though I say okay. My brain says okay, but my heart still no, it's not okay. (Yasmin)

Participants described suppressing their feelings and views to avoid conflict and appeasing employers by apologising even when they felt they were not in the wrong. This undermined their sense of agency and reinforced the perception that their feelings and judgements were less important or valid, ultimately eroding their self-worth. As Laura reflected, “I don't want to have problems with the employers, so I'm trying to keep just giving. Always giving and giving, but always giving it's not good.”

Conversely, participants described that employers who provided support when they faced personal difficulties helped them feel worthy of kindness, which encouraged self-compassion.

It's really helped me. I can become a better person, love myself more, take good care of myself and my health. They also teach me how to save more money that next time when things goes wrong, [I] can stand on [my] own. (Sheryl)

#### **Family obligations and self-care**

The participants described feeling obligated to prioritise providing for their families over their own needs. This sense of duty made practising self-compassion challenging as they often struggled with guilt and self-blame while trying to balance their personal needs with the pressure of being financial providers.

Sometime family know [I’m] here working, so they think I have more money, they know only money, then I don't give them because I also want to keep my money when I go back later. So they want to ask right now I cannot give them so they talk bad about me. I not good. I'm no good daughter, I'm no good [neighbour], I no good cousin for them. But I really do this one for them. (Ruth)

Participants further stated that living apart from their families led to feelings of self-blame and guilt for not being present for important moments. These emotions reinforced self-critical narratives, diminishing their sense of self-worth, and making it more challenging to practice self-compassion. As Sheryl shared, “I so useless you know, when she [mother] not well I’m not around. I always working for money. Working here is only get money but we don’t have the family love.”

#### **Marginalisation and self-worth**

As highlighted in the preceding subthemes, participants faced various stressors that negatively impacted their well-being due to their marginalised status as MDWs. Consequently, they reported feelings of helplessness, sadness, stress, and anxiety, along with behavioural challenges, such as excessive eating, tearfulness, rumination, and patterns of negative thinking, including suicidal ideation. Some participants emphasised the importance of self-compassion given their isolation and distance from support systems back home, viewing it as key to maintaining independence and responsibility. As Laura shared, “I'm working here, I’m by myself. So if anything happened to me, no one helping me. So you have to get up. I mean, [tell myself] you can do it. You can't just give up and do nothing.”

Some participants feared the consequences of voicing the challenges they faced as marginalised individuals.

I even asked MOM [Ministry of Manpower] officer before, will [participation in a media interview] affect my work permit? You’re calling me to come here in your office? And I’m glad she said no, it won’t. Just want to make it clear. I didn’t say anything bad about MOM or about the employer. I told what’s my experience all about. I didn’t criticise anyone. (Janelle)

This fear highlighted a tension in practising self-compassion; while speaking up was identified as a form of self-compassion in an earlier subtheme, the broader context often discouraged it by rendering such actions risky.

Additionally, the participants noted that their MDW status often led others to look down on them, fostering a sense of inferiority and unworthiness that undermined their ability to practice self-compassion.

Even though we are work as a domestic helper or whatever, but we're the same, you know, we are human. We are still a human. I hear also some story from my other friend once saying how their employer treat them is not right. So I think you have to respect yourself, you have to fight for your own right too, like for this thing. Like you know how to show yourself that no one can bullying you or kind of thing because of your job. And it's very common here. People look down on us because we are doing this job. And sometime they think that we don't know anything (…) So I think it is how to show yourself you not just helper (…) and to tell [inaudible] to love yourself, like respect yourself not to let other make you down. (Yasmin)

As marginalised individuals, MDWs often experience challenging working conditions. For participants recognising this reality became a foundation for their practice of self-compassion, particularly by comparing their situations with those of other MDWs and realising that they were relatively better off. This perspective helped normalise their struggles and reinforced their gratitude for their circumstances.

Sometimes simple sharing, that [they] don't have enough food, might be the elderly that they are looking after very noisy, always screaming or even trying to beating them because they [have] dementia they don’t know what right. So I will comparing this to myself that I have better place. I have better condition from them. So I become not so bad. I have more than what they have. (Rosalind)

Overall, this theme situated self-compassion within the structural realities of migrant domestic work, illustrating how self-compassion developed in tension with dependency, obligation and marginalisation.

### Enacting self-compassion in everyday life

This theme captured how participants enacted self-compassion in their everyday lives, through emotional regulation, behavioural care and relational support. These expressions of self-compassion showcased it as a set of intentional, situated practices woven into their general coping and meaning making.

#### **Emotional and cognitive resilience**

Participants shared that acknowledging their stress and adopting a healthy approach by refraining from placing additional pressure on themselves, letting go of unnecessary stress, and reminding themselves not to overextend was an approach to practising self-compassion.

I don't want to put too much burden on myself. I know is my work not easy. So I have to be lightened myself to not thinking too much about other thing that unnecessary (…) I will more take it easy. I lighten myself. I don't want to put so much thing on myself. (Rosalind)

Participants expressed that cultivating a positive mindset promoted self-compassion by enabling them to approach adversity with hope. As Sheryl described, “I don’t want to keep [thinking] about the sickness, worry about thing whatever no good negative thing. I try to keep a positive mindset.” Some participants noted that practising gratitude helped them do so.

Make sure that be more grateful so I know I’m practising this like before I sleep thinking about some blessings that I got, simple for example all my laundry dry because it was sunny, or it’s good to be rain today, I can sleep well. (Melissa)

Some participants described how accepting difficult circumstances helped them practice flexibility and adapt to challenges by making peace with them.

When we acceptance, I think it’s very important. Because if we don’t accept, we end up complain and we keep asking why, why like this, why like that. And there’s no answer you know. Some of thing don’t have answer. (Melissa)

Most participants identified positive self-talk as a key strategy for practising self-compassion. This included encouraging, reassuring, and comforting internal dialogue during difficult times, which was practised through daily affirmations. As Ruth shared, “I support myself. I talk in in the mirror. It's okay. They don't know about you. You are pretty, you're good enough. You hardworking. You good people.” Some participants mentioned that avoiding personalising difficulties while normalising mistakes and challenges helped reduce their feelings of self-criticism. As Melissa shared, “If I want to cry, I will just cry. If feel this is not okay, then it’s okay to be not okay.”

Participants explained that when they recognised themselves entering a self-critical mindset, they consciously redirected their thinking by drawing on more encouraging and realistic appraisals. Yasmin noticed herself slipping into self-blame and countered this by recalling past successes, normalising the situation and reminding herself that she is capable. This deliberate reframing represents a self-compassionate effort to respond to difficulty with encouragement rather than criticism.

So, I think … sometime that I can't do it. But again, my brain said no, you can do it, it's okay. Remember the other time you [did] it, you know you have, you know and [in] fact everything is back to normal. Just think, this thing also, happen the same way. Just keep that again I say. Maybe the most important for me is how to react [to] the situation that will happen [to] me again. Because I keep blaming myself (…) So I will think or do something that make me happy. This is how I try to be kind to myself. (Yasmin)

Other participants reframed negative thoughts by rationalising job-related trade-offs, which helped them reduce frustration and view their sacrifices as meaningful. For example, Rosalind shared her perspective on the trade-off between time off and financial compensation.

So, [at] first is very difficult. But I look at myself again that I've been in Singapore for many years. So I will telling myself, enough time to play. I really explored a lot of places in Singapore. I've done marathon. So I will call myself to be enough. Enough so it's okay, now this job, give me more salary than before. So I will look at that point. So I become more flexible towards my job. (Rosalind)

Many participants described self-compassion as involving patience and gentleness with themselves in the face of challenges. This included taking breaks from work, allowing time to process difficult emotions, and moving at a pace that felt manageable. As Frieda shared, “I'm ready to help myself. I give myself time to heal, time to be alone. I said (.) just give me time, then when I'm ready… the good thing can make me happy.”

Such practices reflected an intentional effort to respond to difficulties with kindness, which helped cultivate emotional and cognitive resilience. In contrast, moments of self-criticism, self-blame, or self-doubt, pulled participants away from a self-compassionate stance and reinforced feelings of unworthiness, which made it even harder for them to care for themselves. As Sheryl shared, “I sometimes blaming myself so why I work so hard for what? In the end, I got nothing.” Together these accounts illustrate the dynamic movement between self-compassionate responses and more critical inner dialogue that shaped how participants understood and enacted self-compassion.

#### **Behavioural strategies**

Most of the participants described self-care as an essential form of self-compassion. Engaging in hobbies and activities such as exercise, sports, shopping, listening to music, writing, watching TV shows, walking in nature, eating well, going for massages, reading, showering, journaling, singing, and taking time for rest were mentioned as ways to improve mood and support well-being. Some participants viewed self-care as a reward for facing challenges that fostered a deeper sense of self-worth and self-compassion.

Sometimes I will reward myself with online shopping. Sometimes I will be buying what I like, especially when I'm going out. Whatever I like to eat, I will eat, or I don't care about the cost. That techniques to treat myself. I must get a reward after very hard working (… ) I call it a compassion that I rewarding myself. (Rosalind)

Some participants described faith and prayer as foundations of self-compassion that provided relief and hope during difficult times, by fostering emotional resilience. As Sheryl shared, “pray is the most important, because this one is the basic and the foundation… we know if we don’t pray, it’s very difficult to control our mind.”

Some participants also reported practising calming strategies as a form of self-compassion. These included grounding techniques, such as breathing, drinking water, and seeking a quiet place.

Self-compassion meaning for me is about how I respond to bad things or bad times. I can respond with calm and just stay cool. If cannot handle it, maybe just drink some water, something that can help me calm down. The thing is how we respond and react. (Melissa)

#### **Engaging social support**

Most participants highlighted engaging social support in order to practice self-compassion. Confiding in trusted peers and even employers during difficult times normalised and validated their struggles, reinforcing their self-worth and helping them view themselves as deserving of self-compassion. As Ruth shared, “Because I have a lot grumble my heart. Nobody listened, and when I meet friend, I’m happy they listen, they understand me. The more people understand me, I’ll make myself more kind [to myself].”

Engaging social support not only provided participants with advice on practising self-compassion, but also modelled self-compassionate self-talk.

After I’m talking with them, I feel relieved, and they supported me with positive words. They are so kind to me. From that, I try to put all those positive words to myself. That’s how I become kind to myself. (Hannah)

Collectively, these practices reflect participants’ definitions of what it meant to be kind to oneself, illustrating how self-compassion is enacted amid challenging work and living conditions.

Across all themes, self-compassion emerged as a dynamic process which was negotiated within the constraints of migration, family obligation and employment. The results demonstrate that the practices that nurtured self-worth and self-compassion were continually negotiated against the structural pressures that eroded that worth and constrained the practice of self-compassion. They also highlight the ongoing tension between self-sacrifice and self-care.

## Discussion

This qualitative study explored the perceptions and experiences of self-compassion among MDWs in Singapore, generating three themes that examined how they conceptualised and enacted self-compassion within the contextual challenges of dependent and restrictive employment conditions, familial obligations and migration-related marginalisation. On the surface, the research findings reveal that participants strove to develop a sense of purpose and identity beyond work, build supportive relationships with peers and employers, and engage in resilience-building practices. Together this fostered self-compassion by reinforcing self-worth, aligning with existing research on resilience and coping, which underscores the value of social support and adaptive strategies in enhancing MDWs’ well-being (Van Bortel et al., 2019; van der Ham et al., 2014; Zainal & Barlas, 2022). Consistent with existing research suggesting that certain marginalised groups may cultivate self-compassion to cope with systemic challenges (Vigna et al., 2018), the participants in this study drew upon internal and relational resources that reinforced their sense of dignity and personal value.

More fundamentally, the findings indicate that self-compassion was activated, shaped, and constrained by the structural contexts of challenging employer relationships, marginalised status, and family obligations, echoing Parreñas and colleagues' research on the structural precarity of migrant domestic work (Parreñas & Silvey, 2018; Parreñas et al., 2021b). Participants’ accounts referred to limited earning opportunities in home countries, restrictive employment contracts and dependency upon employers which often prompted them to suppress their own needs in order to endure hardship and fulfil family obligations.

Given this contextual backdrop, the themes intersected at points and occasionally sat in tension. For example, participants experienced a bi-directional relationship between caring for themselves and caring for their families. Providing for their families enhanced their sense of self-worth and at the same time the pressure of family demands left them feeling guilty and undermined self-worth. Self-compassion was therefore essential for maintaining the strength demanded by their caregiving roles, yet it was often their sense of responsibility toward family that became a reason to practise self-compassion in the first place. This interplay created a tension in which family obligations could both activate and drain their need for self-kindness.

While Neff's (2003a) six-factor model provides a useful conceptual reference for understanding the multidimensional nature of self-compassion, the intention of this analysis was not to fit participants’ experiences into a pre-existing framework. Rather, comparison highlights the points of convergence and divergence between MDWs’ lived expressions of self-compassion and how the construct is commonly measured in the literature. In doing so, the aim is to show how MDWs’ understanding of self-compassion is situated within their sociocultural realities, rather than assuming that Neff’s model fully aligns with their conceptualisations.

A key divergence from Neff’s view (Neff, 2003a, 2003b) was the finding that participants grounded their practice of self-compassion in a foundational sense of self-worth. Neff’s conceptualisation of self-compassion does not involve evaluations of self-worth but rather entails approaching a constantly evolving sense of self with kindness and understanding, especially in moments of perceived personal inadequacy. Instead, the findings align with other research showing that recognising self-worth is crucial for developing self-compassion (Donald et al., 2018; Gilbert et al., 2011) and that individuals who feel vulnerable or hold negative self-views often struggle to practice self-compassion as they perceive themselves as undeserving (Gilbert et al., 2011; Pauley & McPherson, 2010). The centrality of self-worth, and its vulnerability to relational and structural pressures, was reflected across the components of Neff’s conceptualisation of self-compassion.

### **Self-kindness**

Participants conceptualised self-compassion as practising self-kindness by defending their rights, prioritising personal needs, and setting boundaries in challenging situations. This included leaving difficult environments, cultivating a positive mindset, and engaging in self-care. Participants appeared to find these self-compassionate acts easier when their self-worth was robust. However, practising self-kindness often sat in tension with structural conditions.

When harsh treatment undermined their self-worth, participants found it harder to practise self-kindness. Furthermore, owing to dependency on employers and obligations towards family members, participants sometimes felt unable to act in the very ways they defined as self-kindness; they could not speak up for themselves, or they chose to prioritise their families’ needs over their own. This tension between self-sacrifice and self-care aligns with Parreñas (2015).

Participants also described practising self-kindness through positive self-talk, gratitude, prayer, being patient with themselves, and using calming techniques. These practices align with Neff’s view of self-kindness as the adoption of a supportive attitude during difficult times. Beyond Neff’s model, yet in line with studies from Philippines and Indonesia (Concha, 2025; Kotera et al., 2022), self-compassion appeared to include a relational component. By seeking social support and receiving compassion from others, participants not only felt more deserving of self-kindness but also encountered interpersonal models upon which to base more compassionate self-talk.

### **Self-judgement**

Participants’ pride in their ability to endure challenging contextual circumstances in order to meet family obligations provided a foundation for a strong sense of self-worth and, in turn, positive self-judgement. However, the act of providing for their families financially simultaneously required prolonged separation which often prompted self-critical narratives, underpinned by guilt and self-blame. This negative self-talk in turn reinforced their negative self-perceptions. These experiences align with Neff’s concept of self-judgement as an uncompassionate response to oneself through harsh evaluation of perceived inadequacies. In this context, however, perceived inadequacies were less an outcome of personal failure and more shaped by dependency, control, and ‘soft violence’ embedded in MDWs’ sociocultural positioning (Parreñas et al., 2021a).

### **Common humanity**

Neff’s concept of common humanity involves recognising personal failures, mistakes, and suffering as universal aspects of human experience. On the one hand, by acknowledging their marginalisation participants were able to practice self-compassion by drawing comparisons with other MDWs. Recognising that some peers faced even more adverse conditions helped normalise their struggles as part of a collective journey, reducing feelings of isolation, and allowing them to reframe challenges as shared experiences rather than personal failings. On the other hand, their experiences of marginalisation and indentured labour also eroded a sense of common humanity. Being monitored or treated as inferior contributed to a belief that they were fundamentally less worthy, different from others and personally responsible for the hardships they faced, reinforcing guilt and self-blame. In these moments, participants’ capacity to access common humanity was contingent upon relationships in which they were recognised as equals, typically with other MDWs, and undermined in contexts where their value and dignity was persistently denied such as within employer households or migration systems that rendered their presence temporary or disposable (Parreñas et al., 2021b).

### **Isolation**

Participants reported experiences of being looked down upon and feeling marginalised within the community. While they did not explicitly label these experiences as isolation, exclusion operates as a form of isolation, which can lead to feelings of inferiority and unworthiness, fostering the perception that their challenges were their fault (Anjara et al., 2017). Furthermore, employer restrictions often created and maintained tangible isolation; withholding rest days or access to mobile phones isolated participants from families back home and from peer networks in Singapore. Experiencing a sense of common humanity, as described above, depends on being able to connect and share experiences with others who occupy similar positions. When such connections are restricted, participants are denied access to the relational spaces in which they feel equal, valued, and understood. In this way, isolation not only hinders common humanity but also the forming and maintenance of relationships that might restore dignity and affirm self-worth.

### **Mindfulness**

Consistent with Neff’s concept of mindfulness, the participants described cultivating awareness and acceptance of stressful circumstances. They practised mindful awareness by acknowledging their difficulties, responding constructively, and letting go of unnecessary pressure. Acceptance of challenges enabled them to approach difficulties flexibly and make peace with them. Participants also managed stress by reframing negative thoughts with balanced perspectives, such as rationalising work-related trade-offs and time off for financial remuneration. These enactments of self-compassion arose out of their challenging and precarious working conditions. On the one hand such practices are adaptive and allowed MDWs to cope in situations where speaking up conflicted with their desire to provide for their families, and perhaps even constituted small acts of resistance against structural devaluation (Parreñas, 2015). On the other hand, these practices risk being structurally reinforced, normalising emotional suppression and self-silencing, and thus using self-compassion to maintain unequal power relations and convince MDWs that their own needs are unimportant.

### **Over-identification**

Participants described how criticism from employers often led them to internalise blame and question their self-worth, making it difficult to view themselves as deserving of kindness. They also noted how obligatory pressure to send money to their families back home intensified feelings of guilt and self-blame. Consistent with Neff’s concept of over-identification, this absorption of routine disrespect and excessive control eroded their sense of self-worth and heightened distress. The resulting, structurally driven internalisation of inferiority compromised their capacity for self-compassion, as participants struggled to detach from self-critical narratives and adopt a more compassionate view of their experiences. This process operated as a mechanism through which structural devaluation undermined the self-worth participants identified as central to practising self-compassion, and was reinforced by employer relationships that rarely provided the recognition or equality needed to counter self-critical thinking.

Overall, these findings enrich existing research by illustrating how MDWs conceptualisations and enactments of self-compassion are contextually negotiated and not merely individually defined. This aligns with research indicating that sociocultural background can influence the perception of self-compassion (Montero-Marin et al., 2018). These results echo Southeast Asian research showing that self-compassion in collectivist contexts is often embedded in relational obligations and communal identities (Concha, 2025; Kotera et al., 2022). Furthermore, it draws links with research on the structural precarity of migrant domestic work to demonstrate how dependency and obligations fundamentally shape how self-worth is formed and sustained and how self-compassion is enacted (Parreñas et al., 2021a, 2021b).

## Limitations

To address potential limitations in participants’ understanding, a brief introduction to self-compassion was included in the interview schedule (see Appendix A), without providing them with Neff's (2016) conceptualisation. This decision was made considering the often-lower educational levels and English as a second language for most MDWs. While this may have primed participants to some extent, the responses they gave extended beyond the brief introduction, which did not detail Neff’s full conceptualisation. It is also acknowledged that conducting interviews in participants’ first languages may have resulted in different, potentially language-based, conceptualisations of self-compassion.

Another limitation was the study’s self-selecting sample, which consisted of relatively experienced MDWs with at least eight years of work experience in Singapore. These participants may have more established support networks and developed adaptive coping strategies that have influenced their perceptions and experiences of self-compassion. Therefore, the findings may not represent younger MDWs with less experience who may lack similar coping resources. The requirement for English proficiency may have further reduced participation from MDWs facing language barriers who could be more vulnerable to isolation and exploitation (Anjara et al., 2017). Finally, MDWs without rest days or internet access would have been unable to participate. Although the research aimed to explore cultural differences in self-compassion, focusing on Indonesians and Filipinos did not reveal significant differences in their experiences.

## Future research

Further research could benefit from a more diverse sample, including MDWs of different nationalities, backgrounds, ages, and experience levels. Longitudinal studies exploring the development of self-compassion and other coping strategies among MDWs could provide valuable insights into critical periods of vulnerability throughout their migratory journeys as well as how their experiences and resilience evolve over time. Addressing language barriers by conducting research in participants’ first language could provide more nuanced insights. Given the unique experiences and perceptions of self-compassion of the MDWS highlighted in this study, there may be a need for more culturally sensitive measures to assess self-compassion in minority groups that may not be captured by existing tools such as the SCS. Thus, future research could explore the cross-cultural validation of the SCS or the development and validation of more contextually and culturally responsive measures of self-compassion for minority groups. Finally, given that past research (e.g. HOME, 2015) shows how MDWs’ mental health and, as demonstrated in this study, their sense of self-worth appeared contingent on how they are treated by their employers, future research could explore employer attitudes and behaviours towards MDWs.

### Clinical implications

These findings can inform the development of contextually responsive self-compassion interventions tailored to MDWs. These interventions could include self-help resources and psychoeducational materials that emphasise building self-worth as a foundation for self-compassion, recognising that marginalised individuals may require additional support to see themselves as deserving of compassion. These findings could also inform contextually responsive clinical interventions targeted at improving coping and resilience among MDWs. These interventions could incorporate elements addressing cultural values around family obligations and balancing them with self-care, while integrating mental, emotional, and behavioural strategies that have been identified to shape practice of self-compassion.

Finally, it is critical to avoid framing a structurally produced problem as an individual mental health deficit. Clinicians working with MDWs should attend to the ways structural and migration-related conditions influence their mental health, especially their sense of self-worth, and the extent to which these conditions shape and constrain the practice of self-compassion.

## Conclusion

This study revealed that self-compassion among MDWs is a multidimensional construct influenced by both internal perceptions and broader sociocultural influences. Participants’ perceptions and experiences of self-compassion were closely tied to their self-concept and continually negotiated within the structural conditions of migrant domestic work. Experiences of marginalisation, dependency upon employers and transnational family obligations influenced how participants viewed their own worth and the extent to which they felt able to extend compassion to themselves. At the same time, relationships of mutual recognition, with peers or, in some cases, employers, along with efforts to develop purpose and identity beyond work and resilience-building practices, supported the restoration of dignity and self-worth.

At a societal level, these findings underscore the need to address structural determinants of MDWs well-being. Advocating for policy reforms to protect the rights of MDWs and for employer education focused on their ethical treatment is essential to creating fair labour conditions in which self-compassion can develop alongside dignity and self-worth, not merely be used as a coping strategy within exploitative systems. Such initiatives could be facilitated by the Ministry of Manpower through targeted programmes and regulatory oversight.

## Supplementary Material

Supplementary material MDW self compassion supplementary.

## Data Availability

Data can be obtained from the corresponding author upon reasonable request.
