# Supplementary material for: Self-compassion in context: a reflexive thematic analysis of migrant domestic workers’ experiences in Singapore
Source: Int J Qual Stud Health Well-being. 2026 Jan 19;21(1):2617449. doi: 10.1080/17482631.2026.2617449 (PMC12818332; doi:10.1080/17482631.2026.2617449)
Supplement: Supplementary material — MDW self compassion supplementary. [file ZQHW_A_2617449_SM6803.docx]

# **Semi-Structured Interview Schedule**

**Opening**

Thank you for agreeing to be a part of this study on self-compassion among helpers in Singapore. Today, I would like to ask you some questions about what you think about self-compassion, and if you could share any personal experience about practicing self-compassion while living and working in Singapore in your role as a helper. I hope to use this information to gain a better understanding about how helpers in Singapore think about and experience self-compassion. The interview should take about an hour. Would that be alright with you?

**General demographic information**

- Which country are you from?
- How long have you been working in Singapore?
- How old are you?

**General Questions on Self-Compassion**

Today, I would like to ask you some questions about self-compassion. To some people, self-compassion means being supportive towards yourself when you are experiencing suffering or pain, whether this is in response to challenges you have faced, caused by personal mistakes, or thinking you’re not good enough.

***Conceptualization of self-compassion***

1. What does self-compassion mean to you?

2. Would you have any different words/ways to describe self-compassion?

3. Do you have any personal experiences practicing self-compassion while living and working in Singapore? If so, could you describe that to me?

- Can you describe a difficult situation that you’ve been in during your time living and working here in Singapore? In what ways was your reaction to this self-compassionate/not self-compassionate? (to be used as a prompt if necessary)

4. Can you think of any benefits to practicing self-compassion in your work and life here in Singapore?

5. Can you think of any down-sides to practicing self-compassion in your work and life here in Singapore?

***Barriers and facilitators in practicing self-compassion***

6. What makes it easier for you to practice self-compassion in your work and life here in Singapore?

7. Is there anything that gets in the way of you being able to practice self-compassion in your work and life here in Singapore?

8. What would help you practice self-compassion more in the future?

9. Could you think of any opposite to (self-compassion)? And if so, what would that be?

10. Can you think of any benefits to (the opposite of self-compassion)?

***Closing Questions***

Thank you for sharing your thoughts and experiences with me.

- Are there any other ideas or experiences you want to share that we have not touched on which you think could be helpful?
- Do you have any thoughts about what we have discussed today or any other thoughts about self-compassion amongst helpers living and working in Singapore?

Once again, thank you very much for your time.
